# Diet Quality and Sleep Characteristics in Midlife: The Bogalusa Heart Study

**DOI:** 10.3390/nu15092078

**Published:** 2023-04-25

**Authors:** Kaitlin S. Potts, Maeve E. Wallace, Jeanette Gustat, Sylvia H. Ley, Lu Qi, Lydia A. Bazzano

**Affiliations:** 1 Department of Epidemiology, Tulane University School of Public Health and Tropical Medicine, New Orleans, LA 70112, USA; gustat@tulane.edu (J.G.); sley@tulane.edu (S.H.L.); lqi1@tulane.edu (L.Q.); lbazzano@tulane.edu (L.A.B.); 2 Division of Sleep and Circadian Disorders, Department of Sleep Medicine, Brigham and Women’s Hospital, Harvard Medical School, Boston, MA 02115, USA; 3 Department of Social, Behavioral and Population Sciences, Tulane University School of Public Health and Tropical Medicine, New Orleans, LA 70112, USA; mwallace@tulane.edu; 4 Department of Nutrition, Harvard T.H. Chan School of Public Health, Boston, MA 02115, USA

**Keywords:** diet quality, dietary patterns, sleep apnea, sleep disordered breathing, insomnia, sleep quality

## Abstract

Background: Sleep and diet contribute to cardiometabolic disease, but evidence is sparse for the association between these behaviors. This study analyzed the cross-sectional relationship between diet quality and multiple sleep outcomes in the Bogalusa Heart Study (BHS). Methods: Diet and sleep characteristics, including insomnia and sleep apnea symptoms, were measured with validated questionnaires. Poisson regression using generalized estimating equations with a log link estimated prevalence rate ratios (PRR) of sleep outcomes by dietary pattern scores (quintile (Q) and per SD). Models were adjusted for body mass index (BMI), multi-level socioeconomic factors, physical activity, depressive symptoms, and other potential confounders. Results: In 824 participants, higher diet quality, measured by the Alternate Healthy Eating Index-2010, was associated with lower sleep apnea risk score after adjustment (PRR [95% confidence interval (CI)] Q5 vs. Q1: 0.59 [0.44, 0.79], per SD increase: 0.88 [0.81, 0.95], *p*-trend < 0.0001). There were no statistically significant associations with the Healthy Eating Index 2015 or the Alternate Mediterranean dietary patterns, or for insomnia symptoms or a healthy sleep score. Conclusions: Higher diet quality, after adjustment for BMI, was associated with a lower sleep apnea risk score in a cohort with substantial minority representation from a semi-rural, lower-income community.

## 1. Introduction

Cardiovascular disease (CVD) remains the leading cause of death in the U.S. and affects 49% of adults [1,2]. Lifestyle behaviors, including sleep and diet, are modifiable risk factors for CVD and remain suboptimal for much of the population [2,3,4]. Fewer than half of American adults meet any dietary recommendation made by the American Heart Association [3], yet higher adherence to a healthy diet may confer 21% risk reduction for CVD [5]. At the same time, many Americans experience inadequate sleep. Nearly 35% habitually sleep less than the recommended 7 h nightly [6,7], up to 30% suffer from insomnia [8], and 34% may have moderate to severe sleep apnea [9]. Insomnia and sleep apnea have consistently been associated with increased risk of CVD [4].

Much research has focused on the relationship of these behaviors to health outcomes, but evidence is sparse on the association between diet and sleep. Short-term clinical trials support the hypothesis that there is a cyclical relationship between sleep and diet, with sleep influencing next-day eating behavior and diet affecting sleep through metabolic pathways [10]. Over the longer term, nutrient deficiencies and anthropometry influence sleep disorders [11,12,13]. Some studies have examined the association of overall diet quality measured by dietary pattern indices (e.g., the Alternate Healthy Eating Index, AHEI) on sleep outcomes prospectively [14,15] and cross-sectionally [16,17,18,19,20,21,22], finding that higher diet quality often associates with better sleep outcomes. However, the totality of evidence is minimal, many of these studies were conducted in non-diverse cohorts (e.g., all men, all women, Mediterranean cohorts), and most have assessed a single dimension of sleep, such as sleep duration, with one measure of diet quality. Further understanding of how diet quality influences sleep could improve primordial prevention of CVD and other diseases with clinical applications in improving behavioral interventions targeting these behaviors. Even small improvements in such interventions could have a substantial public health impact in long-term reductions in chronic diseases. In addition, both sleep and diet are influenced by numerous structural, social, and environmental factors [23,24,25,26] and exhibit differences by sex, race, and socioeconomic status [2,4], lending support to their interplay as a potential contributor to population health inequities, adding to the public health relevance of this topic.

The objective of this study was to explore the cross-sectional relationship between diet quality and multiple sleep outcomes, including insomnia, sleep apnea, and a healthy sleep pattern in a biracial (Black and White) population of adult men and women in the Bogalusa Heart Study (BHS) and to determine if these effects differed by sex, race, and socioeconomic status. We hypothesized that higher diet quality would be inversely associated with poor sleep outcomes. This report expands on a brief presentation of these results in conference proceedings [27].

## 2. Materials and Methods

### 2.1. Study Design and Population

**Figure 1:**
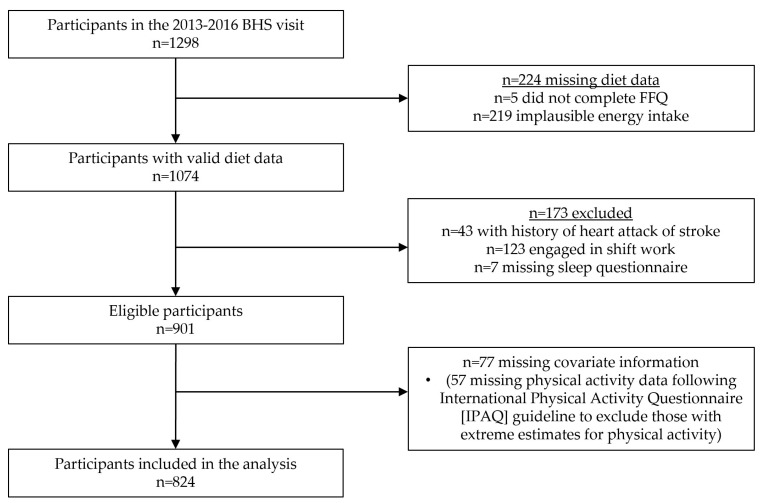
Participant flowchart.

The BHS is a community-based, semi-rural cohort of predominantly Black and White persons from Bogalusa, Louisiana that began in 1972 [28]. Repeated assessments of individuals have continued to the present day. Participants from the 2013–2016 visit, which included a comprehensive sleep questionnaire and dietary assessment, were eligible for this cross-sectional study. All participants gave written, informed consent prior to data collection and study procedures were approved by the Tulane University Health Sciences Institutional Review Board. Among 1298 participants in the 2013–2016 BHS survey, those with implausible energy intake were excluded (*n* = 224, Figure 1). Additional excluded participants were those with a history of heart attack or stroke (*n* = 43), engaged in shift work (*n* = 123), missing data on diet or sleep (*n* = 7), or missing covariate information (*n* = 77, most due to implausible physical activity data). The final sample size included in this analysis was 824.

### 2.2. Measures

#### 2.2.1. Dietary Assessment

Diet was measured with the Lower Mississippi Delta Nutrition Intervention Research Initiative (Delta NIRI) Food Frequency Questionnaire (FFQ). The Delta NIRI is a 142-item quantitative FFQ developed and tested for validity and reproducibility among individuals in the Jackson Heart Study as a regionally specific tool [29]. It includes food items culturally relevant for BHS participants such as okra, grits, crawfish, gumbo, and jambalaya which are not included in most other FFQs developed for general U.S. populations. The Delta NIRI measures usual intake and includes questions about the frequency of consumption (from never to 6+ times per day) and typical portion size (four food-appropriate choices) for each item. The Delta NIRI was validated against four 24-hour recalls (two weekdays and two weekends) over six months [30]. Correlation coefficients comparing nutrient estimates to the means of the recalls were similar to those found in validations of several other FFQs (e.g., correlation coefficient was 0.70 for carbohydrates and 0.54 for saturated fat) [30]. Delta NIRI foods were matched to foods in the USDA Food Patterns Equivalent Database 2015–2016 [31] to estimate intake of 37 food groups. Nutrient intakes were estimated using the Nutrient Database for Scientific Research [32]. As noted above, individuals with implausible energy intake, defined during the Delta NIRI validation as <600 or >4800 kcal/day, were excluded [29,30].

Three dietary pattern scores were calculated. Higher scores indicate closer adherence to pre-defined diets. The Healthy Eating Index (HEI) 2015 measures adherence to the 2015 Dietary Guidelines for Americans [33]. The Alternate Healthy Eating Index (AHEI) 2010 reflects a diet that directly incorporates the evidence between diet and health [34]. The Alternate Mediterranean Dietary Pattern (aMed) was developed to measure adherence to a Mediterranean-type diet focused on plant foods and proteins, monounsaturated fats, and fish with reduced intakes of saturated fats and animal foods [35]. Both the HEI and the AHEI components are scored based on specific intake values, although the HEI uses energy-adjusted intakes, while the aMed components are scored based on sex-specific median cutoffs. See Supplementary Table S1 for details on scoring each dietary pattern. The HEI-2015 ranges from 0 to 100, the AHEI-2010 ranges from 0 to 111, and the aMed ranges from 0 to 9. Dietary pattern scores were grouped into quintile groups to reduce the impact of outliers, minimize the influence of measurement error in the FFQ, and to provide meaningful comparison groups while not assuming a linear relationship. Quintiles were chosen in lieu of quartiles for the utility of having a middle group to capture the middle range of diet quality while providing greater differentiation compared to using tertiles. Dietary patterns were also used continuously in the analysis.

#### 2.2.2. Measurement of Sleep Outcomes

Multiple sleep domains were measured with a self-report questionnaire at the same time as the dietary assessment. The Women’s Health Initiative Insomnia Rating Scale (WHIIRS) is a valid and reliable 5-item scale measuring insomnia risk (see Table S2) [36]. The items ask about the frequency during the past four weeks of four insomnia symptoms (trouble falling asleep, night waking, waking too early, and trouble getting back to sleep) and the quality of a typical night’s sleep. A score >9 indicates high risk for insomnia as validated in multiple populations compared to objective measures [36,37].

The Berlin Questionnaire is a 10-item, 3-category tool validated to classify individuals as high risk for sleep apnea (see Table S3) [38]. The three categories assess snoring, sleepiness, and presence of high blood pressure or obesity. A positive score in two or more categories indicates high risk for sleep apnea, and has been shown to be valid (86% sensitive, 77% specific) for identifying those with an apnea–hypopnea index >5, a clinical cutoff for mild sleep apnea [38].

Overall sleep health was captured with the healthy sleep pattern, a newly developed score that associates with lower risk of CVD in the U.K. Biobank and the China Kadoorie Biobank [39]. The score considers five domains of sleep including chronotype, sleep duration, insomnia symptoms, snoring, and daytime sleepiness and ranks each as healthy or unhealthy. Early chronotype, 7–8 hours’ sleep duration, and infrequent insomnia symptoms, snoring, and daytime sleepiness are scored as healthy. The five domains are summed (healthy = 1, unhealthy = 0) to obtain an overall score (0–5), where ≥4 indicates a healthy sleep pattern. Measurement tools for each domain included the reduced Morningness-Eveningness Questionnaire for chronotype [40], self-report weekday and weekend sleep duration, the WHIIRS for insomnia symptoms [37], the Berlin Questionnaire for snoring [38], and the Epworth Sleepiness Scale for daytime sleepiness [41].

#### 2.2.3. Measurement of Covariates

Demographic, socioeconomic, health, and lifestyle factors were also assessed. Age, sex, education, employment status, having a bed partner, household size, number of children in the house, smoking status, current alcohol use, and illicit drug use were collected with questionnaires. Race was based on self-report and all participants included in the present analysis identified as Black or White persons. Race is not used in this study as the basis for identifying potential biological differences, but as a marker of the impact of structural racism that has been embedded in our society for centuries and has been recognized as the underlying cause of many racial health inequities. Physical activity was measured with the International Physical Activity Questionnaire (IPAQ) and assesses activity in all domains of daily living (occupational, domestic, recreational, and walking) [42]. Total metabolic equivalent of task (MET)-minutes per week was calculated after summing frequency and duration of each activity and applying activity-specific MET values. Per the IPAQ scoring guidelines, those who overreported hours per week of activity were excluded. The 10-item Centers for Epidemiologic Studies Depression (CESD) scale measured depressive symptoms (score ≥10) [43]. Weight and height were measured in duplicate, and averages were taken to calculate BMI as kg/m^2^.

Neighborhood (census tract) factors were measured by geocoding residential addresses to latitude and longitude coordinates using the MMQGIS plugin in QGIS [44,45]. Those that did not match were hand-searched in Google Maps; *n* = 19 remained missing. Address coordinates were spatially joined to a census tract shape file with Federal Information Processing codes for merging with data on census tract-level indicators obtained from the American Community Survey (ACS) 2013 5-year estimates [46]. To further capture racial- and economic-related disadvantage beyond what is captured by individual-level factors such as race and education, the Index of Concentration at the Extremes (ICE) was calculated. The ICE measures racial and economic segregation based on neighborhood race and income distributions [47]. The ICE was calculated with 2013 ACS 5-year data as the number of White householders with ≥ USD 100,000 annual income (the privileged group) minus the number of Black householders with < USD 25,000 annual income (the disadvantaged group), divided by the total number of households reporting income in the tract. The ICE ranges from -1 to 1, where values below 1 indicate greater numbers in the disadvantaged compared to the privileged group in the area. The Modified Retail Food Environment Index (mRFEI) is the percentage of total food retailers in the tract that are considered healthy and was used a measure of food environment quality [48].

### 2.3. Statistical Analysis

Descriptive statistics are presented for the total sample and by quintiles of AHEI-2010. ANOVA and Pearson chi-squared tests assessed differences across quintiles. Multivariable Poisson regression using generalized estimating equations (GEE) with log link estimated prevalence rate ratios (PRRs) for sleep outcomes for each quintile of dietary patterns compared to Q1, and per standard deviation (SD) increase in dietary pattern scores. Models accounted for clustering at the census tract level. Trends across quintiles were tested by assigning the median dietary pattern score in each quintile and treating these as continuous variables. Effect modification was tested by assessing the *p*-value from interaction terms in adjusted models, and by stratifying the results where relevant.

Potential confounders were identified a priori based on a hypothesized causal structure and associations identified in the literature. If potential confounders were highly correlated (R > 0.7), selection was based on improved model fit. Results are given for three successive nested models adjusting for demographic, socioeconomic, health, and lifestyle factors. Model 3 included total energy intake (kcal/d), age (y), sex, race (Black or White), education (no college, any college or more), employment (not employed, any employment), bed partner (yes, no), number of children in house, census tract ICE, total number of households in census tract, census tract mRFEI, smoking status (never, current, former), drinking status (non-drinker, current drinker), caffeine intake (mg/d), illicit drug use (any current use, none), frequent sleeping pill use (3+ times/w, <3 times/w), depressive symptoms (yes, no), BMI (kg/m^2^), and physical activity (MET-minutes/d). As mentioned above, those with cardiovascular events were excluded, but additional subclinical cardiovascular comorbidities were not included in the adjusted models as they were hypothesized to play a larger role in this causal structure as colliders, downstream of both sleep and diet. Data cleaning and analysis were conducted in R, version 4.0, and SAS, version 9.

Several sensitivity analyses were performed. Drinking status was removed from the models with AHEI and aMed since these include an alcohol component. We tested the removal of BMI from the models for sleep apnea since the Berlin questionnaire includes BMI >30 kg/m^2^ as a component. Finally, we performed the analysis after removal of those with depressive symptoms and those who frequently use sleeping pills, separately.

## 3. Results

The mean age of participants was 48 (± 5.2) years, 36% were male and 30% were of Black race (Table 1). Nearly 30% of the sample suffered from depressive symptoms and more than half were persons with obesity. A total of 44% had a high risk for insomnia and 44% had a high risk for sleep apnea, while 23% had a healthy sleep pattern. The mean AHEI-2010 score was 45.2 (see Table S4 for distribution of other dietary patterns). Those in higher quintiles of AHEI-2010 were more often older (*p* = 0.002), female (*p* = 0.049), higher educated (*p* = 0.004), never smokers (*p* < 0.0001), alcohol drinkers (*p* < 0.0001), and not depressed (CESD-10 <10) (*p* = 0.004) (Table 1; comparisons by HEI and aMed are in Table S5; comparisons by sleep outcomes in Table S6).

Those who were excluded from the sample because of missing data on exposure, outcome, or covariates were more likely to be male, Black persons, have less education, not have a bed partner, live in areas with lower ICE, currently smoke, have higher physical activity, and have depressive symptoms (Table S7). There were no significant differences in age, employment status, dietary pattern scores, or sleep outcomes between the included and excluded groups.

Being at high risk for insomnia was inversely associated with AHEI-2010, HEI-2015, and aMed in the unadjusted model and after adjustment for sociodemographic factors (Table 2). These associations were no longer statistically significant in Model 3, except for Q5 vs. Q1 of the aMed score, but neither the test for trend nor the per-SD effect was statistically significant.

Being at high risk for sleep apnea was inversely associated with AHEI-2010 and HEI-2015 but not the aMed in the unadjusted model; the association with AHEI-2010 remained significant in Model 3. Compared to Q1, those in Q5 of AHEI-2010 were 41% less likely to be at high risk for sleep apnea (PRR [95% CI] = 0.59 [0.44, 0.79]). There was a significant trend across quintiles (*p* for trend < 0.0001), and the association was significant when AHEI-2010 was treated as a continuous variable with 12% lower prevalence of high sleep apnea score per 10-point increase in AHEI-2010 (PRR [95% CI] per SD [10.0] increase: 0.88 [0.81, 0.95]). Table S8 shows the association with the sub-components, snoring and sleepiness, of the Berlin Questionnaire. Being positive on the sleepiness component but not the snoring component was associated with higher AHEI-2010.

Having a healthy sleep pattern was not associated with any of the dietary patterns except for a marginal association with AHEI-2010 in the unadjusted model (Table 2). In addition, none of the individual components (Table S9), nor the full score (range of 0–5 treated as a continuous outcome, results not shown) of the healthy sleep pattern were associated with AHEI-2010.

Tests for interaction between AHEI-2010 and sex, race, and education are given in Table 3. The association between AHEI-2010 and sleep apnea risk was stronger among women than men (PRR [95% CI] per SD increase in women: 0.80 [0.74, 0.88], in men: 1.00 [0.88, 1.13], *p*-value for interaction per SD increase = 0.004, *p*-value for interaction between trend variables = 0.028). This association was also stronger among those with more education (PRR [95% CI] per SD increase in high education group: 0.83 [0.75, 0.92], in low-education group: 0.97 [0.88, 1.08], *p*-value for interaction per SD increase = 0.004, *p*-value for interaction between trend tests = 0.009). There were no statistically significant interactions between AHEI-2010 and race nor between HEI-2015 or aMed and sex, race, or education.

To determine which components of AHEI-2010 contribute most to the association with sleep apnea risk, we tested this association with each of the 11 AHEI components (Table 4). Fruits, long-chain (n-3) fatty acids (EPA + DHA), sugar-sweetened beverages, red and processed meats, and alcohol had significant inverse associations (higher scores for all components indicate healthier diet pattern—i.e., adverse food groups are already inversed, see Table S1) in Model 3 when comparing Q5 to Q1, and all but the fruits component were also statistically significant when treated continuously.

None of the sensitivity analyses meaningfully changed the results. Supplementary Tables S10 and S11 show the results after removal of those with depressive symptoms and frequent sleeping pill use, respectively.

## 4. Discussion

We observed an inverse association between diet quality and being at high risk for sleep apnea in this cross-sectional study. Higher diet quality measured by AHEI-2010 was associated with lower prevalence of high risk for sleep apnea measured by the Berlin Questionnaire. Those in Q5 of AHEI-2010 had 41% lower prevalence of high sleep apnea risk compared to those in Q1, and each 10-point increase in AHEI-2010 related to 12% lower prevalence, after adjustment for socio-demographic, health, and lifestyle factors. This association was stronger among women and in those with more education. We did not find any statistically significant associations between diet quality and being at high risk for insomnia or having an overall healthy sleep pattern, nor were there associations with HEI-2015 or aMed dietary patterns.

Similar results were identified using objective measures of sleep apnea in a cross-sectional Multi-Ethnic Study of Atherosclerosis (MESA) study [9]. Those with apnea–hypopnea index ≥15 from in-home polysomnography, indicating moderate to severe sleep apnea, had lower diet quality measured by AHEI-2010, and had higher intake of red and processed meats. Although we did not use objective measures, these results confirm their findings and extend them to those with mild, moderate, or severe sleep apnea as measured by an easy-to-use clinical assessment tool, the Berlin Questionnaire.

Others have found higher overall diet quality associated with better sleep outcomes other than sleep apnea. Multiple cross-sectional studies have observed shorter sleep duration among those with lower diet quality, including in the MESA cohort [18], the Women’s Health Initiative study [16], female Iranian university students [49], Australian women [22], Italian school children [50], and a Hispanic cohort [17]. In multiple Mediterranean cohorts, higher adherence to a Mediterranean diet associated with better sleep quality, cross-sectionally [20,21] and prospectively [14]. Unlike those studies, we did not find any significant associations with aMed. This could be due to diet differences between populations, since the aMed score is based on median intakes within the population in which it is scored. In contrast to our study, insomnia symptoms were associated with diet quality in a prospective analysis in the Health Professionals Follow-up Study [51], cross-sectionally in the MESA cohort, and among elderly women [18,19].

The HEI-2015 has not been studied in relation to sleep outcomes as often as Mediterranean dietary patterns, but it is interesting that no associations were observed for this pattern in the present study. There are multiple differences between the AHEI-2010 and the HEI-2015 dietary patterns that may explain these findings (Table S1 provides a summary of differences). One potential source may be the different treatment of fruit juice. Fruit juice is included in the total fruits component of HEI-2015 and counted as a positive food item, whereas it is included in the sugar-sweetened beverages and fruit juice component of AHEI-2010, which is scored as a negative component (inversed so lower intakes score higher). This seems like an important difference since this AHEI-2010 component was statistically significantly associated with sleep apnea risk in the components analysis (Table 4). Other differences may also be important, such as the lack of a dairy component in AHEI-2010 or the different treatment of fatty acids between these dietary patterns, but further research is needed to explore the sources of these differential findings and to identify what dietary pattern(s) are most relevant for improving sleep-related outcomes.

We identified effect modification by sex and education. There was a stronger relationship between AHEI-2010 and sleep apnea risk in women and in those with more education, although limited sample sizes may have impacted the ability to detect an effect in the smaller strata. Women may be less likely to have sleep apnea than men [52], but the sample of men in our study was relatively small. Among those with high school education or less, higher diet quality did not relate to lower prevalence of sleep apnea risk, suggesting multifactorial influences coinciding with socioeconomic disadvantage. We did not identify an interaction by race. More Black participants were at high risk for sleep apnea than White participants (50% compared to 42%), but there was no statistically significant difference in diet quality by race. In addition, similar proportions were exposed to low-quality diets among those with sleep apnea risk in both Black and White participants.

Diet quality may influence sleep through a number of pathways. The most relevant for sleep apnea risk may be the development of obesity and accumulation of extra tissues around the upper airway, a mechanical mechanism linking high BMI to sleep apnea [53]. Young et al. estimated that 41% of U.S. cases of mild, moderate, or severe sleep apnea may be attributable to excess body weight [53]. Sleep apnea risk defined by the Berlin Questionnaire includes obesity as a component, so it is not surprising that diet quality plays a role in being at high risk [38]. Although the cross-sectional design of this study limits our ability to interpret potential pathways, it seems unlikely that obesity alone is sufficient to explain the observed association between diet quality and sleep apnea risk. This is supported by the sensitivity analysis where the removal of BMI from the final model did not alter the results. Other mechanisms linking diet to sleep include metabolism and absorption of specific nutrients; e.g., dietary tryptophan coupled with carbohydrate consumption and insulin release promotes the synthesis of melatonin and serotonin, essential regulators of the sleep–wake cycle [10]. Nutrient deficiencies may also influence sleep (e.g., Vitamin D) [11]. It is unclear if and how these other pathways relate to sleep apnea specifically, but it is feasible that there are multiple mechanisms linking diet quality and the risk of sleep disordered breathing.

This study has many strengths. The population is a Black and White cohort from a lower-income, semi-rural community in the southeastern U.S., where health inequities are prevalent. Validated, reliable instruments were used to measure insomnia and sleep apnea risk and a culturally specific, validated FFQ assessed usual diet. These tools allow for comparability with other epidemiologic studies and clinical settings. We controlled for many potential confounders, including frequent sleeping pill use, depressive symptoms, and neighborhood-level factors, allowing more control of confounding by socioeconomic conditions than other similar studies accounted for. The robustness of the results was tested with multiple sensitivity analyses, and we tested for interactions by race, sex, and education.

Some limitations also apply. The cross-sectional nature of our study does not allow causal inference or interpretation of the directionality of the relationship between diet quality and sleep apnea, but it is relevant for generating hypotheses to be tested with prospective designs. The use of subjective questionnaires to measure diet and sleep introduces potential measurement errors; however, these tools were validated, have been widely used, and subjective sleep measures have strong relevance to patient quality of life [54]. Small sample sizes may have limited our power to detect statistically significant effects between diet quality and insomnia risk and to detect interactions. Although we controlled for many potential confounders at multiple levels, observational studies cannot eliminate the possibility of residual confounding. For example, self-report measurement of physical activity, as performed in this study with the IPAQ, is particularly challenging and prone to measurement error [42]. Finally, those who were excluded differed from the participants included in the analysis in some ways, reducing generalizability. In addition, this sample represents a lower-income, semi-rural, Black and White population; generalizability to other populations needs to be confirmed in studies among other cohorts.

This study identified an association between higher diet quality and decreased prevalence of high risk for sleep apnea. Future prospective observational studies are needed to confirm the presence of and elucidate the directionality of this relationship. Although we did not detect an association between diet quality and insomnia risk or healthy sleep pattern, additional studies with larger sample sizes should be conducted to confirm this. Understanding the relationship between diet and sleep could improve programs and policies aimed at risk reduction of CVD and other chronic diseases. If diet is an important contributor to sleep outcomes, sleep interventions may be more effective by incorporating diet quality components. In addition, if future studies confirm specific components of a healthy diet that are most relevant for improved sleep, these could inform recipes and menus of diet interventions targeting overall sleep quality.

## Figures and Tables

**Table 1:** Description of participants in the total sample and by quintile of the Alternate Healthy Eating Index 2010: the Bogalusa Heart Study 2013–2016.

	Total Sample ^a^	Quintile of Alternate Healthy Eating Index-2010 ^b^			*p*-Value ^c^
		Q1	Q3	Q5	
	*n* = 824	*n* = 163	*n* = 166	*n* = 169	
Demographic characteristics					
Age in years	48.29 ± 5.18	47.44 ± 5.31	48.43 ± 5.21	49.23 ± 4.80	0.002
Male (%)	36.17	35.58	31.93	29.59	0.049
Black persons (%)	29.85	24.54	37.35	27.98	0.113
Years of education (%)					
Less than high school	10.56	14.72	11.45	5.92	0.004
High school	34.71	39.26	38.55	26.63	
Some college or higher	54.73	46.01	50.00	67.46	
Employed (%)	63.35	58.90	60.24	68.64	0.300
Bed partner (%)	62.26	62.58	55.42	63.91	0.220
Household size, persons	2.89 ± 1.45	2.86 ± 1.36	2.99 ± 1.44	2.80 ± 1.24	0.602
Number of children in the house	0.85 ± 1.08	0.86 ±1.02	0.95 ± 1.20	0.76 ± 0.99	0.174
Neighborhood characteristics (census tract)					
ACS: % persons in poverty	26.29 ± 10.96	27.11 ±9.70	27.08 ±10.99	22.92 ± 12.01	0.0002
ACS: median income	36,364.08 ± 17,408.19	34,595.61 ± 17,450.58	34,879.18 ± 16,114.36	42,479.30 ± 21,679.29	<0.0001
ACS: % households with no vehicle	10.36 ± 6.69	10.93 ± 6.78	10.44 ± 6.24	8.90 ± 6.74	0.019
ACS: Index of Concentration at the Extremes	−0.05 ± 0.20	−0.05 ± 0.17	−0.08 ± 0.20	0.00 ± 0.21	0.001
ACS: total households	1749.99 ± 775.36	1727.52 ± 1035.20	1740.71 ± 669.04	1834.49 ± 785.86	0.310
Modified retail food environment index	11.60 ± 9.16	11.32 ± 9.63	11.71 ± 9.20	11.36 ±8.99	0.953
Health and lifestyle factors					
Smoking status (%)					
Never	53.88	47.24	57.23	63.91	<0.0001
Former	22.45	16.56	15.66	26.63	
Current	23.67	36.20	27.11	9.47	
Current alcohol use (%)	56.07	42.94	46.99	77.51	<0.0001
Total energy intake, kcal/d	2319.30 ± 1019.58	2660.54 ± 901.85	2070.36 ± 998.06	2057.09 ± 934.93	<0.0001
Caffeine intake, mg/d	257.84 ± 329.26	296.17 ± 346.17	220.87 ± 312.87	229.10 ± 260.86	0.173
Physical activity, MET minutes per week	4941.33 ± 5135.69	4285.45 ± 4755.41	4996.31 ± 5688.94	5827.31 ± 5348.46	0.079
Illicit drug use (%)	33.62	42.33	29.52	30.77	0.053
Frequent sleeping pill use (%)	17.84	19.02	22.89	14.20	0.217
Depressive symptoms (%)	28.28	36.20	32.53	19.53	0.004
CESD-10 score	7.44 ± 5.53	8.23 ± 6.03	7.94 ± 5.55	6.02 ± 4.45	0.0004
Body mass index, kg/m^2^	31.37 ± 7.64	31.05 ± 6.73	32.27 ± 8.41	30.27 ± 7.62	0.159
Obesity (%)	52.18	57.06	53.01	44.38	0.198
Waist circumference, cm	96.02 ± 19.01	95.79 ± 16.64	97.14 ± 19.82	92.20 ± 20.84	0.047
Sleep outcomes					
High risk for insomnia (%)	44.05	51.53	44.58	36.09	0.029
High risk for sleep apnea (%)	44.05	52.76	50.00	30.77	0.0006
Healthy sleep pattern (%)	22.94	19.63	19.88	29.59	0.175

^a.^ % or mean ±SD among total sample. ^b.^ % or mean ±SD among column total (total in quintile); Q2 and Q4 are not shown for brevity. ^c.^ *p*-value from comparison across all quintiles: ANOVA for continuous variables and Pearson chi-squared for categorical variables. AHEI: Alternate Healthy Eating Index. ACS: American Community Survey, 2013 5-year estimates. MET: Metabolic equivalent of task. CESD-10: Center for Epidemiologic Studies Depression Scale—Revised, 10-item. Employed includes full- or part-time employment. Depressive symptoms defined as CESD-10 ≥ 10. Frequent sleeping pill use defined as 1–2 times per week or more. Obesity defined as body mass index ≥ 30 kg/m^2^. High risk for insomnia defined as > 9 on the Women’s Health Initiative Insomnia Rating Scale. High risk for sleep apnea defined as positive in two of three categories on the Berlin questionnaire. Healthy sleep pattern determined if healthy pattern in three of five sleep domains: chronotype, duration, insomnia symptoms, snoring, and daytime sleepiness.

**Table 2:** Prevalence rate ratios for sleep outcomes by quintile and per standard deviation increase in dietary pattern scores.

	Model	Q1	Q2 ^a^	Q3 ^a^	Q4 ^a^	Q5 ^a^	*p* for Trend ^b^	Per SD Increase ^a^
Alternate Healthy Eating Index 2010								
Q *n* (median)	163 (32.52)		162 (39.41)	166 (44.32)	164 (50.36)	169 (57.91)		SD = 10.01
Insomnia risk	1	1.00	0.95 (0.75, 1.20)	0.87 (0.73, 1.03)	0.77 (0.64, 0.93) **	0.70 (0.52, 0.94) *	0.0004	0.88 (0.82, 0.95) ***
	2	1.00	1.03 (0.83, 1.27)	0.91 (0.74, 1.10)	0.83 (0.67, 1.04)	0.75 (0.57, 0.99) *	0.009	0.90 (0.83, 0.97) **
	3	1.00	1.06 (0.87, 1.29)	0.90 (0.70, 1.15)	0.95 (0.78, 1.15)	0.86 (0.67, 1.11)	NS	0.94 (0.87, 1.01)
Sleep apnea risk	1	1.00	0.83 (0.67, 1.02)	0.95 (0.79, 1.13)	0.82 (0.69, 0.98) *	0.58 (0.45, 0.76) ***	<0.0001	0.86 (0.80, 0.93) ***
	2	1.00	0.81 (0.65, 1.01)	1.00 (0.85, 1.17)	0.84 (0.72, 0.97) *	0.61 (0.47, 0.79) ***	<0.0001	0.88 (0.82, 0.94) ***
	3	1.00	0.77 (0.61, 0.97) *	0.91 (0.79, 1.05)	0.80 (0.68, 0.95) *	0.59 (0.44, 0.79) ***	<0.0001	0.88 (0.81, 0.95) **
Healthy sleep pattern	1	1.00	1.10 (0.81, 1.49)	1.01 (0.62, 1.66)	1.21 (0.85, 1.72)	1.51 (0.97, 2.34)	0.048	1.15 (1.00, 1.33)
	2	1.00	1.10 (0.82, 1.49)	0.98 (0.61, 1.57)	1.15 (0.80, 1.67)	1.39 (0.92, 2.10)	NS	1.13 (0.98, 1.30)
	3	1.00	1.14 (0.80, 1.64)	0.97 (0.60, 1.55)	1.05 (0.78, 1.41)	1.19 (0.80, 1.77)	NS	1.05 (0.93, 1.19)
Healthy Eating Index 2015								
Q *n* (median)	160 (46.82)		172 (54.39)	165 (58.97)	165 (63.93)	162 (71.04)		SD = 9.17
Insomnia risk	1	1.00	1.03 (0.86, 1.23)	0.88 (0.65, 1.19)	0.94 (0.79, 1.13)	0.72 (0.55, 0.94) *	0.003	0.91 (0.85, 0.98) **
	2	1.00	1.07 (0.88, 1.32)	0.89 (0.67, 1.17)	0.99 (0.80, 1.23)	0.71 (0.55, 0.91) **	0.002	0.91 (0.85, 0.98) **
	3	1.00	1.05 (0.87, 1.26)	0.89 (0.72, 1.10)	1.01 (0.84, 1.23)	0.85 (0.67, 1.10)	NS	0.96 (0.89, 1.03)
Sleep apnea risk	1	1.00	0.91 (0.70, 1.17)	1.03 (0.83, 1.29)	0.88 (0.73, 1.07)	0.76 (0.61, 0.94) *	0.014	0.92 (0.85, 0.98) *
	2	1.00	0.94 (0.73, 1.21)	1.06 (0.84, 1.33)	0.93 (0.76, 1.14)	0.77 (0.62, 0.95) *	0.017	0.92 (0.86, 0.99) *
	3	1.00	0.87 (0.70, 1.09)	1.00 (0.81, 1.23)	0.90 (0.72, 1.12)	0.83 (0.66, 1.05)	NS	0.95 (0.87, 1.01)
Healthy sleep pattern	1	1.00	1.15 (0.62, 2.12)	0.97 (0.58, 1.63)	0.97 (0.61, 1.55)	1.31 (0.84, 2.03)	NS	1.08 (0.97, 1.21)
	2	1.00	1.08 (0.62, 1.87)	0.96 (0.60, 1.55)	0.88 (0.58, 1.33)	1.32 (0.86, 2.02)	NS	1.08 (0.97, 1.21)
	3	1.00	1.13 (0.66, 1.96)	0.94 (0.58, 1.53)	0.85 (0.57, 1.26)	1.07 (0.67, 1.72)	NS	1.02 (0.90, 1.15)
Alternate Mediterranean Dietary Pattern								
Q *n* (median)	201 (2.00)		154 (3.00)	164 (4.00)	132 (5.00)	173 (6.00)		SD = 1.78
Insomnia risk	1	1.00	0.83 (0.67, 1.02)	0.85 (0.64, 1.14)	1.05 (0.91, 1.20)	0.75 (0.62, 0.90) **	0.031	0.94 (0.89, 1.00) *
	2	1.00	0.83 (0.68, 1.03)	0.86 (0.63, 1.17)	1.03 (0.84, 1.27)	0.68 (0.55, 0.85) ***	0.017	0.91 (0.85, 0.99) *
	3	1.00	0.86 (0.73, 1.01)	0.91 (0.69, 1.21)	1.04 (0.81, 1.33)	0.81 (0.66, 0.99) *	NS	0.97 (0.89, 1.04)
Sleep apnea risk	1	1.00	0.99 (0.81, 1.22)	1.08 (0.90, 1.29)	1.20 (1.02, 1.41) *	1.04 (0.84, 1.28)	NS	1.02 (0.96, 1.09)
	2	1.00	0.98 (0.80, 1.19)	1.02 (0.86, 1.21)	1.11 (0.92, 1.33)	0.89 (0.73, 1.08)	NS	0.97 (0.91, 1.03)
	3	1.00	0.95 (0.76, 1.20)	0.95 (0.81, 1.12)	1.02 (0.84, 1.24)	0.94 (0.78, 1.14)	NS	0.98 (0.91, 1.06)
Healthy sleep pattern	1	1.00	1.08 (0.83, 1.42)	1.02 (0.78, 1.33)	0.87 (0.60, 1.28)	0.91 (0.70, 1.19)	NS	0.95 (0.88, 1.03)
	2	1.00	1.09 (0.85, 1.40)	1.02 (0.79, 1.31)	0.87 (0.56, 1.36)	0.99 (0.74, 1.31)	NS	0.97 (0.87, 1.08)
	3	1.00	1.04 (0.81, 1.36)	0.98 (0.78, 1.24)	0.84 (0.51, 1.39)	0.77 (0.59, 1.01)	0.049	0.90 (0.81, 0.99) *

^a.^ Prevalence rate ratio (95% confidence interval). ^b.^ *p*-value from dietary pattern trend variable (assigning median value of quintile to all within each quintile and treating this as a continuous variable). * *p* < 0.05, ** *p* < 0.01, *** *p* < 0.001; NS: *p* > 0.05. Model 1: unadjusted. Model 2: total energy intake, age, sex, race, education (no college, any college or higher), employed (full or part time), bed partner, number of people in house, number of children in house, Index of Concentration at the Extremes (ICE) for census tract, total number of households in census tract. Model 3: Model 2 + modified retail food environment index (mRFEI) for census tract, smoking status (never, current, former), drinking status (current), caffeine intake (mg/d), current illicit drug use (yes/no), frequent sleeping pill use (1–2 times per week or more), depressive symptoms (CESD-10 > 10), body mass index (kg/m^2^), physical activity (total MET-minutes per week).

**Table 3:** Adjusted prevalence rate ratios for sleep outcomes by quintile and per standard deviation increase in Alternate Healthy Eating Index 2010, stratified by sex, race, and education.

AHEI 2010		Q1	Q2 ^d^	Q3 ^d^	Q4 ^d^	Q5 ^d^	*p* for Trend ^e^	Per One SD (10.01) Increase	*p* for Interaction ^f^
Insomnia risk ^a^	Men	1.00	1.12 (0.78, 1.62)	0.81 (0.44, 1.48)	0.97 (0.69, 1.36)	0.91 (0.52, 1.57)	NS	0.96 (0.84, 1.08)	NS
	Women	1.00	1.01 (0.76, 1.34)	0.89 (0.73, 1.09)	0.89 (0.66, 1.19)	0.84 (0.66, 1.07)	NS	0.92 (0.84, 1.01)	
Sleep apnea risk ^a^	Men	1.00	0.76 (0.56, 1.03)	1.11 (0.87, 1.41)	0.81 (0.59, 1.13)	0.78 (0.52, 1.18)	NS	1.00 (0.88, 1.13)	0.028
	Women	1.00	0.76 (0.59, 0.98) *	0.76 (0.60, 0.96) *	0.79 (0.62, 1.01)	0.48 (0.35, 0.66) ***	<0.0001	0.80 (0.74, 0.88) ***	
Healthy sleep ^a^	Men	1.00	1.36 (0.73, 2.53)	1.49 (0.46, 4.84)	1.81 (1.05, 3.14)	1.52 (0.61, 3.83)	NS	1.14 (0.92, 1.42)	NS
	Women	1.00	1.14 (0.67, 1.91)	0.83 (0.53, 1.30)	0.77 (0.51, 1.15)	1.01 (0.68, 1.49)	NS	0.99 (0.87, 1.14)	
Insomnia risk ^b^	Black	1.00	1.40 (1.01, 1.95) *	0.98 (0.62, 1.55)	1.08 (0.61, 1.89)	1.30 (0.82, 2.04)	NS	1.03 (0.92, 1.15)	NS
	White	1.00	1.00 (0.78, 1.27)	0.89 (0.74, 1.08)	0.96 (0.76, 1.21)	0.77 (0.57, 1.05)	NS	0.91 (0.83, 1.00)	
Sleep apnea risk ^b^	Black	1.00	1.00 (0.68, 1.47)	0.96 (0.60, 1.54)	0.78 (0.47, 1.30)	0.77 (0.37, 1.57)	NS	0.90 (0.72, 1.12)	NS
	White	1.00	0.70 (0.51, 0.94) *	0.92 (0.76, 1.11)	0.86 (0.71, 1.04)	0.54 (0.39, 0.74) ***	<0.0001	0.88 (0.81, 0.95) **	
Healthy sleep ^b^	Black	1.00	1.43 (1.04, 1.96)	1.50 (0.74, 3.03)	1.51 (0.52, 4.32)	1.71 (0.93, 3.12)	NS	1.11 (0.87, 1.40)	NS
	White	1.00	1.15 (0.72, 1.83)	0.87 (0.51, 1.49)	1.05 (0.73, 1.52)	1.16 (0.72, 1.88)	NS	1.08 (0.93, 1.25)	
Insomnia risk ^c^	Low ed.	1.00	1.03 (0.83, 1.28)	0.95 (0.71, 1.27)	1.04 (0.83, 1.29)	0.85 (0.59, 1.23)	NS	0.95 (0.87, 1.04)	NS
	High ed.	1.00	1.06 (0.83, 1.35)	0.81 (0.59, 1.13)	0.84 (0.61, 1.16)	0.90 (0.67, 1.22)	NS	0.93 (0.84, 1.04)	
Sleep apnea risk ^c^	Low ed.	1.00	0.73 (0.56, 0.94) *	0.90 (0.71, 1.14)	0.86 (0.66, 1.13)	0.86 (0.61, 1.22)	NS	0.97 (0.88, 1.08)	0.009
	High ed.	1.00	0.80 (0.58, 1.11)	0.95 (0.72, 1.26)	0.79 (0.59, 1.06)	0.50 (0.34, 0.72) ***	0.0001	0.83 (0.75, 0.92) ***	
Healthy sleep ^c^	Low ed.	1.00	1.37 (0.92, 2.04)	1.22 (0.77, 1.93)	1.16 (0.62, 2.17)	1.35 (0.74, 2.47)	NS	1.01 (0.85, 1.20)	NS
	High ed.	1.00	0.95 (0.51, 1.77)	0.79 (0.45, 1.39)	0.95 (0.58, 1.54)	1.02 (0.63, 1.64)	NS	1.08 (0.93, 1.26)	

^a.^ Sex stratified analysis included 298 men and 526 women. ^b.^ Race stratified analysis included 246 Black participants and 578 White participants. ^c.^ Education stratified analysis included 373 participants with low education (high-school degree or less) and 451 participants with high education (some college or more). ^d.^ Prevalence rate ratio (95% confidence interval). ^e.^ *p*-value from dietary pattern trend variable (assigning median value of quintile to all in each quintile and treating this as a continuous variable) within strata of effect modifier. ^f.^ *p*-value from product-term between dietary pattern trend variable and effect modifier. * *p* < 0.05, ** *p* < 0.01, *** *p* < 0.001; NS: *p* > 0.05. Models adjusted for the following, except sex, race, and education, were removed when stratified by those variables: total energy intake, age, sex, race, education (no college, any college or higher), employed (full or part time), bed partner, number of children in house, Index of Concentration at the Extremes (ICE) for census tract, total number of households in census tract, modified retail food environment index (mRFEI) for census tract, smoking status (never, current, former), drinking status (current), caffeine intake (mg/d), current illicit drug use (yes/no), frequent sleeping pill use (1–2 times per week or more), depressive symptoms (CESD-10 > 10), body mass index (kg/m^2^), and physical activity (total MET-minutes per week).

**Table 4:** Adjusted prevalence rate ratios for high risk for sleep apnea by components of Alternate Healthy Eating Index 2010 dietary pattern (*n* = 824).

Component of AHEI-2010 Dietary Pattern	Q1	Q2 ^a^	Q3 ^a^	Q4 ^a^	Q5 ^a^	*p* for Trend	Per One Unit Increase ^a^
1. Fruits	1.00	0.90 (0.78, 1.04)	0.90 (0.74, 1.09)	0.95 (0.79, 1.15)	0.77 (0.60, 1.00) *	NS	0.97 (0.95, 1.00)
2. Vegetables (no potatoes)	1.00	0.87 (0.72, 1.05)	0.93 (0.74, 1.18)	1.18 (1.00, 1.40)	0.85 (0.68, 1.07)	NS	1.00 (0.98, 1.02)
3. Nuts and legumes	1.00	1.28 (1.02, 1.61) *	1.15 (0.91, 1.45)	1.04 (0.85, 1.27)	1.16 (0.94, 1.42)	NS	1.00 (0.97, 1.04)
4. Whole grains	1.00	1.03 (0.83, 1.28)	1.20 (0.87, 1.65)	0.87 (0.65, 1.14)	1.03 (0.77, 1.38)	NS	0.99 (0.96, 1.03)
5. Long-chain (n-3) fats	1.00	0.75 (0.57, 0.98) *	0.88 (0.74, 1.05)	0.81 (0.68, 0.97) *	0.70 (0.50, 0.98) *	NS	0.97 (0.95, 1.00) *
6. Polyunsaturated fats	1.00	1.02 (0.74, 1.40)	0.94 (0.73, 1.19)	0.88 (0.62, 1.24)	0.96 (0.68, 1.36)	NS	0.99 (0.95, 1.03)
7. Sugar-sweetened beverages + fruit juice ^b^	1.00	0.83 (0.69, 0.98) *	0.87 (0.75, 1.00)	0.75 (0.60, 0.93) *	NA	0.010	0.98 (0.96, 0.99) **
8. Red and processed meats ^b^	1.00	0.89 (0.68, 1.17)	0.87 (0.70, 1.07)	0.73 (0.55, 0.96) *	NA	0.017	0.97 (0.94, 1.00) *
9. *Trans* fats	1.00	0.98 (0.84, 1.16)	0.97 (0.84, 1.11)	0.91 (0.74, 1.11)	0.98 (0.67, 1.45)	NS	0.97 (0.93, 1.01)
10. Sodium	1.00	0.99 (0.84, 1.18)	1.02 (0.82, 1.27)	1.05 (0.84, 1.31)	1.06 (0.72, 1.56)	NS	1.00 (0.97, 1.04)
11. Alcohol	1.00	0.91 (0.68, 1.22)	0.95 (0.73, 1.24)	1.00 (0.79, 1.28)	0.76 (0.64, 0.91) **	0.008	0.98 (0.95, 1.00) *

^a.^ Prevalence rate ratio (95% confidence interval). ^b.^ There are only 4 groups (quartiles) for sugar-sweetened beverages and red and processed meats. * *p* < 0.05, ** *p* < 0.01; NS: *p* > 0.05. Models adjusted for total energy intake, age, sex, race, education (no college, any college or higher), employed (full or part time), bed partner, number of children in house, Index of Concentration at the Extremes (ICE) for census tract, total number of households in census tract, modified retail food environment index (mRFEI) for census tract, smoking status (never, current, former), drinking status (current), caffeine intake (mg/d), current illicit drug use (yes/no), frequent sleeping pill use (1–2 times per week or more), depressive symptoms (CESD-10 > 10), body mass index (kg/m^2^), and physical activity (total MET-minutes per week).

## Data Availability

The data presented in this study are available on request from the corresponding author.

## Supplementary Materials

The following supporting information can be downloaded at: https://www.mdpi.com/article/10.3390/nu15092078/s1. Table S1: Components and scoring of dietary patterns Alternate Healthy Eating Index 2010, Healthy Eating Index-2015, and Alternate Mediterranean Diet Score; Table S2: Women’s Health Initiative Insomnia Rating Scale; Table S3: Berlin questionnaire for sleep apnea risk; Table S4: Description of dietary patterns; Table S5: Description of participants by dietary patterns Healthy Eating Index 2015 and Alternate Mediterranean Diet Score; Table S6: Description of participants by insomnia risk, sleep apnea risk, and healthy sleep pattern; Table S7: Comparison of those included in the analysis with those not meeting inclusion criteria or excluded due to missing on covariates; Table S8: Adjusted prevalence rate ratios for components of Berlin Questionnaire for sleep apnea risk by quintile and per standard deviation increase in Alternate Healthy Eating Index 2010 dietary pattern; Table S9: Adjusted prevalence rate ratios for healthy classification of each component of the Healthy Sleep Pattern by quintile and per standard deviation increase in Alternate Healthy Eating Index 2010 dietary pattern; Table S10: Adjusted prevalence rate ratios for sleep outcomes by quintile and per standard deviation increase in Alternate Healthy Eating Index 2010 dietary pattern, among those without depressive symptoms (CESD-10 < 10). (*n* = 591); Table S11. Adjusted prevalence rate ratios for sleep outcomes by quintile and per standard deviation increase in Alternate Healthy Eating Index 2010 dietary pattern, among those who do not use sleeping pills frequently (< once per week). (*n* = 677).
