# Supplementary material for: Diet Quality and Sleep Characteristics in Midlife: The Bogalusa Heart Study
Source: Nutrients. 2023 Apr 25;15(9):2078. doi: 10.3390/nu15092078 (PMC10180575; doi:10.3390/nu15092078)
Supplement: Supplementary file 1 [file nutrients-15-02078-s001.zip › nutrients-2340602-supplementary.pdf]

Supplemental Material

Table S1. Components and scoring of dietary patterns Alternate Healthy Eating Index 2010 [1], Healthy Eating Index-2015 [2], and Alternate Mediterranean Diet Score [3].

	**AHEI-2010**		**HEI-2015**		**aMed**	
**Summary**	11 components<br>Total score: 0-110	Components score 0-10 between the criteria for min and max score.	13 components<br>Total score: 0-100	Components score 0-5 or 0-10 between the criteria for min and max score.	9 components<br>Total score: 0-9	Components score 0-1 based on sex-specific medians
	**Component**	**Scoring**	**Component**	**Scoring**	**Component**	**Scoring**
Higher intake → higher score	1. Fruits<br>*1 serving =1 medium piece or 0.5 cup berries*	0: 0 servings<br>10: ≥ 4 servings	1. Total fruits (including 100% juice)	0: no fruit<br>5: ≥ 0.8 cup per 1000 kcal	1. Fruit	0: < median<br>1: > median
	2. Vegetables (not potatoes)<br>*1 serving=0.5 cups vegetables or 1 cup greens*	0: 0 servings<br>10: ≥ 5 servings	2. Whole fruits	0: no whole fruit<br>5: ≥ 0.4 cup per 1000 kcal	2. Vegetables (not potatoes)	0: < median<br>1: > median
	3. Nuts and legumes<br>*1 serving=1 oz nuts or 1 tbsp nut butter*	0: 0 servings<br>10: ≥ 1 servings	3. Total vegetables (incl. legumes)	0: no vegetables<br>5: ≥ 1.1 cup per 1000 kcal	3. Nuts	0: < median<br>1: > median
	4. Whole grains	0: 0 grams<br>10: men → ≥ 90 grams<br>women → ≥ 75 grams	4. Greens and beans	0: no dark greens or legumes<br>5: ≥ 0.2 cup per 1000 kcal	4. Legumes	0: < median<br>1: > median
	5. Long chain (n-3) fatty acids (EPA + DHA)	0: 0 mg<br>10: ≥ 250 mg (~2-4 servings of fish per week)	5. Whole grains	0: no whole grains<br>10: ≥ 1.5 oz per 1000 kcal	5. Whole grains	0: < median<br>1: > median
	6. PUFAs	0: ≤ 2% of energy<br>10: ≥ 10% of energy	6. Dairy	0: no diary<br>10: ≥ 1.3 cup per 1000 kcal	6. Fish	0: < median<br>1: > median
			7. Total protein foods	0: no protein foods<br>5: ≥ 2.5 oz per 1000 kcal	7. Fatty acids ratio: (MUFAs/ SFAs)	0: < median<br>1: > median
			8. Seafood and plant proteins	0: no seafood or plant proteins<br>5: ≥ 0.8 oz per 1000 kcal		

			9. Fatty acids ratio	0: (PUFAs + MUFAs) / SFAs ≤ 1.2<br>10: (PUFAs + MUFAs) / SFAs ≥ 2.5		
Lower intake → higher score	7. Sugar sweetened beverages and fruit juice	0: ≥ 1 serving<br>10: 0<br>*1 serving=8 oz*	10. Refined grains	0: ≥ 4.3 oz per 1000 kcal<br>10: ≤ 1.8 oz per 1000 kcal	8. Red and processed meats	0: > median<br>1: < median
	8. Red and processed meats	0: ≥ 1.5 servings<br>10: 0 servings<br>*1 serving=4 oz unprocessed meat or 1.5 oz processed meat*	11. Added sugars	0: ≥ 26% of energy<br>10: ≤ 6.5% of energy		
	9. *trans* fats	0: ≥ 4% of energy<br>10: ≤ 0.5% of energy	12. Saturated fats	0: ≥ 16% of energy<br>10: ≤ 8% of energy		
	10. Sodium	0: highest decile<br>10: lowest decile	13. Sodium	0: ≥ 2.0 grams per 1000 kcal<br>10: ≤1.1 grams per 1000 kcal		
Moderate intake → highest score	11. Alcohol	0: men → ≥ 3.5 drinks<br>women → ≥ 2.5 drinks<br>10: men → 0.5-2.0 drinks<br>women → 0.5-1.5 drinks			9. Alcohol	0: men→ <10 or >25 grams<br>women→ <5 or >15 grams<br>1: men→10-25 grams<br>women→ 5-15 grams

All amounts are intakes per day unless otherwise specified. AHEI: Alternate Healthy Eating Index. HEI: Healthy Eating Index. aMed: alternate Mediterranean. PUFA: polyunsaturated fatty acids. MUFA: monounsaturated fatty acids. SFA: saturated fatty acids.

Table S2. Women's Health Initiative Insomnia Rating Scale [4].

Question (In the last 4 weeks…)	Score) Response options
1. Did you have trouble falling asleep?<br>2. Did you wake up several times at night?<br>3. Did you wake up earlier than you planned to?<br>4. Did you have trouble getting back to sleep after you woke up too early?	0) "no, not in the past 4 weeks"<br>1) "yes, less than once a week"<br>2) "yes, 1 or 2 times a week"<br>3) "yes, 3 or 4 times a week"<br>4) "yes, 5 or more times a week"
5. Overall, was your typical night's sleep during the last 4 weeks:	0) "very sound or restful"<br>1) "sound or restful"<br>2) "average quality"<br>3) "restless"<br>4) "very restless"

Table S3. Berlin questionnaire for sleep apnea risk [5].

Category	Question (In the last 4 weeks)	Response options
Category 1, **Snoring**: this category is positive if the sum of the 5 items is 2 or more	1. Have you snored?	a) Yes (1 point)<br>b) No<br>c) Don't know
	2. Your snoring is:	a) Slightly louder than breathing<br>b) As loud as talking (1 point)<br>c) Louder than talking (1 point)<br>d) Very loud-can be heard in adjacent rooms (1 point)
	3. How often do you snore?	a) Almost every day (1 point)<br>b) 3-4 times/week (1 point)<br>c) 1-2 times/week<br>d) 1-2 times/month<br>e) Never
	4. Does your snoring bother other people?	a) Yes (1 point)<br>b) No<br>c) Don't know
	5. Has anyone ever noticed you stop breathing during your sleep?	a) Almost every day (2 points)<br>b) 3-4 times/week (2 points)<br>c) 1-2 times/week<br>d) 1-2 times/month<br>e) Never

Category 2, **Sleepiness**: this category is positive if the sum is 2 or more	6. How often do you feel tired or fatigued after you sleep?	a) Almost every day (1 point)<br>b) 3-4 times/week (1 point)<br>c) 1-2 times/week<br>d) 1-2 times/month<br>e) Never
	7. During your waking time, do you feel tired, fatigued, or not up to par?	a) Almost every day (1 point)<br>b) 3-4 times/week (1 point)<br>c) 1-2 times/week<br>d) 1-2 times/month<br>e) Never
	8. Have you ever nodded off or fallen asleep while driving?	Yes (1 point)<br>No<br>Don't know
Category 3, **Obesity/ Hypertension**: this category is positive if score is 1 or more	9. BMI > 30 kg/m^2?	Yes (1 point)<br>No
	10. High blood pressure?	Yes (1 point)<br>No

Table S4. Description of dietary patterns.

	**Total sample**		**Q1**	**Q2**	**Q3**	**Q4**	**Q5**
	mean ± SD	median (min, max)	*n* (median)	*n* (median)	*n* (median)	*n* (median)	*n* (median)
AHEI-2010	45.18 ± 10.01	44.37 (17.39, 79.21)	163 (32.52)	162 (39.41)	166 (44.32)	164 (50.36)	169 (57.91)
HEI-2015	59.21 ± 9.17	58.94 (33.71, 87.33)	160 (46.82)	172 (54.39)	165 (58.97)	165 (63.93)	162 (71.04)
aMed	3.92 ± 1.78	4.00 (0.00, 8.00)	201 (2.00)	154 (3.00)	164 (4.00)	132 (5.00)	173 (6.00)

AHEI: Alternate Healthy Eating Index. HEI: Healthy Eating Index. aMed: alternate Mediterranean.

Table S5. Description of participants by dietary patterns Healthy Eating Index 2015 and Alternate Mediterranean Diet Score.

	**HEI [a]**				**aMed [a]**			
	**Q1** *n*=160	**Q3** *n*=165	**Q5** *n*=162	**p value[b]**	**Q1** *n*=201	**Q3** *n*=164	**Q5** *n*=173	**p value[b]**
Demographic characteristics								
Age in years	47.06 ± 5.23	48.81 ± 5.26	49.76 ± 4.51	<0.0001	47.57 ± 5.33	48.34 ± 5.24	48.91 ± 5.03	0.159
Male (%)	41.88	36.36	28.40	0.110	37.31	35.37	32.95	0.862
Black persons (%)	25.63	30.91	31.25	0.681	74.13	67.90	63.01	0.131
Years of education (%)								
Less than high school	12.50	13.94	6.17	0.030	11.94	7.93	10.40	0.092
High school	38.75	35.76	25.93		42.29	32.93	35.26	
Some college and higher	48.75	50.30	67.90		45.77	59.15	54.34	
Employed (%)	55.63	61.21	62.35	0.075	57.21	67.68	61.85	0.053
Bed partner (%)	66.25	64.24	66.05	0.222	64.68	60.98	61.85	0.912
Household size	2.87 ± 1.36	3.04 ± 1.61	2.92 ± 1.44	0.601	2.85 ± 1.43	2.82 ± 1.38	3.08 ± 1.72	0.222
Number of children in the house	0.86 ± 1.08	0.87 ± 1.14	0.87 ± 1.12	0.965	0.91 ± 1.18	0.66 ± 0.92	0.87 ± 1.10	0.098
Neighborhood characteristics (census tract)								
ACS: % persons in poverty	26.90 ± 10.01	26.86 ± 10.78	24.72 ± 11.40	0.240	27.35 ± 10.30	26.71 ± 10.49	25.65 ± 11.45	0.466
ACS: median income	34785.59 ± 15792.85	35635.71 ± 16283.08	38861.80 ± 17175.32	0.200	34782.29 ± 16477.20	35670.73 ± 16905.05	37316.70 ± 17364.95	0.528
ACS: % households with no vehicle	10.46 ± 6.77	10.53 ± 6.40	9.56 ± 6.63	0.510	10.90 ± 6.51	10.29 ± 6.57	10.19 ± 6.77	0.742
ACS: Index of Concentration at the Extremes	-0.05 ± 0.17	-0.06 ± 0.19	-0.03 ± 0.20	0.557	-0.06 ± 0.19	-0.05 ± 0.19	-0.04 ± 0.20	0.949
ACS: total households	1750.16 ± 1044.99	1762.14 ± 734.24	1788.94 ± 655.72	0.908	1757.48 ± 997.52	1748.86 ± 672.72	1767.45 ± 729.89	0.993
Modified retail food environment index	12.14 ± 8.91	12.00 ± 9.30	11.31 ± 9.36	0.535	12.14 ± 9.45	11.19 ± 9.11	12.21 ± 9.15	0.498
Health and lifestyle factors								
Smoking status (%)								
Never	41.25	55.76	64.81	<0.0001	52.74	54.27	61.85	0.005
Former	22.50	20.61	23.46		15.92	21.95	21.97	
Current	36.25	23.64	11.73		31.34	23.78	16.18	
Current alcohol use (%)	46.25	54.55	64.20	0.025	52.24	57.93	60.69	0.176

Total energy intake, kcal/d	2494.26 ± 1019.72	2275.73 ± 1070.64	2175.26 ± 987.30	0.049	1891.60 ± 956.98	2332.01 ± 965.62	2894.38 ± 949.39	<0.0001
Caffeine intake, mg/d	293.84 ± 381.51	253.09 ± 343.93	207.88 ± 215.88	0.178	232.16 ± 281.49	237.57 ± 302.40	266.48 ± 314.07	0.174
Physical activity, MET minutes per week	4348.09 ± 4424.97	5496.95 ± 5966.16	4971.89 ± 4720.02	0.115	3925.61 ± 4482.73	4570.33 ± 5064.09	6099.00 ± 5532.63	0.001
Illicit drug use (%)	40.63	32.12	29.01	0.165	31.84	33.54	28.90	0.373
Frequent sleeping pill use (%)	23.13	18.79	11.11	0.086	22.89	18.29	14.45	0.217
Depressive symptoms (%)	31.25	29.70	16.67	0.002	34.33	26.83	18.50	0.011
CESD-10 score	7.73 ± 5.77	7.80 ± 6.15	5.91 ± 4.27	0.001	8.18 ± 6.33	7.17 ± 5.11	6.37 ± 4.69	0.024
Body mass index, kg/m^2	30.92 ± 7.30	32.02 ± 7.87	30.20 ± 7.28	0.147	30.58 ± 7.21	32.06 ± 8.92	31.27 ± 7.33	0.268
Obesity (%)	54.38	54.55	42.59	0.107	51.24	51.22	47.40	0.397
Waist circumference, cm	95.66 ± 17.90	97.95 ± 18.43	93.11 ± 20.00	0.167	94.33 ± 18.06	96.67 ± 20.46	95.74 ± 19.81	0.299
Sleep outcomes								
High risk for insomnia (%)	48.13	42.42	34.57	0.054	49.25	42.07	36.99	0.048
High risk for sleep apnea (%)	48.13	49.70	36.42	0.124	41.79	45.12	43.35	0.590
Healthy sleep pattern (%)	21.25	20.61	27.78	0.458	23.38	23.78	21.39	0.865

[a.] % or mean ±SD among column total (total in quintile) – Q2 and Q4 not shown for brevity.

[b.] p-value from comparison across all quintiles: ANOVA for continuous variables and Pearson chi-squared for categorical variables.

HEI: Healthy Eating Index. aMed: alternate Mediterranean. ACS: American Community Survey, 2013 5-year estimates. MET: Metabolic equivalent of task.

CESD-10: Center for Epidemiologic Studies Depression Scale Revised 10 item.

Employed includes full or part time employment.

Depressive symptoms defined as CESD-10 ≥ 10.

Frequent sleeping pill use defined as one-two times per week or more.

Obesity defined as body mass index ≥ 30 kg/m^2.

High risk for insomnia defined as > 9 on the Women's Health Initiative Insomnia Rating Scale.

High risk for sleep apnea defined as positive on two of three categories on the Berlin questionnaire.

Healthy sleep pattern determined if healthy pattern on three of five sleep domains: chronotype, duration, insomnia symptoms, snoring, and daytime sleepiness.

Table S6. Description of participants by insomnia risk, sleep apnea risk, and healthy sleep pattern.

	Insomnia risk [a]			Sleep apnea risk [a]			Healthy sleep pattern [a]		
	**Low**	**High**	**p-value [b]**	**Low**	**High**	**p-value [b]**	**No**	**Yes**	**p-value [b]**
	n=461	*n*=363		*n*=461	*n*=363		*n*=635	*n*=189	
Demographic characteristics									
Age in years	47.89 ± 5.34	48.79 ± 4.94	0.014	48.16 ± 5.27	48.45 ± 5.08	0.421	48.39 ± 5.13	47.94 ± 5.36	0.291
Male (%)	39.91	31.40	0.012	32.97	40.22	0.032	36.38	35.45	0.816
Black persons (%)	30.87	28.73	0.506	26.96	33.70	0.036	69.67	71.43	0.643
Years of education (%)									
Less than high school	8.68	12.95	0.013	9.11	12.40	0.304	10.39	11.11	0.815
High school	32.32	37.74		34.92	34.44		35.28	32.80	
Some college and higher	59.00	49.31		55.97	53.17		54.33	56.08	
Employed (%)	71.37	53.17	<0.0001	67.68	57.85	0.004	60.31	73.54	0.001
Bed partner (%)	65.51	58.13	0.030	61.39	63.36	0.562	62.05	62.96	0.820
Household size	2.95 ± 1.44	2.82 ± 1.46	0.173	2.95 ± 1.49	2.83 ± 1.40	0.240	2.88 ± 1.46	2.94 ± 1.43	0.638
Number of children in house	0.91 ± 1.09	0.77 ± 1.06	0.068	0.87 ± 1.11	0.82 ± 1.04	0.514	0.85 ± 1.09	0.83 ± 1.04	0.767
Neighborhood characteristics (census tract)									
ACS: % persons in poverty	25.72 ± 11.53	27.02 ± 10.15	0.092	25.49 ± 11.10	27.31 ± 10.71	0.018	26.62 ± 10.85	25.19 ± 11.29	0.114
ACS: median income	37677.64 ± 18964.34	34661.84 ± 15061.75	0.014	37677.87 ± 18585.37	34661.54 ± 15650.47	0.012	35796.22 ± 16356.20	38206.56 ± 20492.47	0.139
ACS: % households with no vehicle	10.18 ± 6.66	10.59 ± 6.73	0.378	9.99 ± 6.64	10.83 ± 6.73	0.075	10.48 ± 6.57	9.95 ± 7.06	0.341
ACS: Index of Concentration at the Extremes	-0.04 ± 0.21	-0.06 ± 0.18	0.008	-0.03 ± 0.20	-0.07 ± 0.19	0.014	-0.06 ± 0.20	-0.02 ± 0.20	0.044
ACS: total households	1799.11 ± 885.93	1687.61 ± 602.03	0.040	1796.31 ± 861.80	1691.17 ± 645.62	0.046	1749.11 ± 765.20	1752.96 ± 810.63	0.952
Modified retail food environment index	11.99 ± 9.05	11.10 ± 9.30	0.167	11.72 ± 9.02	11.45 ± 9.35	0.675	11.51 ± 9.28	11.89 ± 8.76	0.615
Health and lifestyle factors									
Smoking status (%)									
Never	57.27	49.59	0.022	55.75	51.52	0.096	52.28	59.26	0.045
Former	22.56	22.31		23.43	21.21		22.05	23.81	
Current	20.17	28.10		20.82	27.27		25.67	16.93	
Current alcohol use (%)	59.65	51.52	0.020	54.88	57.58	0.439	55.43	58.20	0.501

Total energy intake, kcal/d	2281.58 ± 1022.97	2367.21 ± 1014.64	0.232	2199.78 ± 1021.24	2471.09 ± 998.41	0.0001	2351.74 ± 1002.27	2210.30 ± 1071.28	0.094
Caffeine intake, mg/d	259.56 ± 316.12	255.65 ± 345.66	0.866	257.67 ± 345.23	258.04 ± 308.26	0.987	254.96 ± 326.98	267.50 ± 337.50	0.646
Physical activity, MET minutes per week	5118.04 ± 5048.83	4716.92 ± 5242.34	0.266	5049.49 ± 5105.44	4803.97 ± 5177.66	0.496	4800.71 ± 2138.26	5413.79 ± 5112.29	0.150
Illicit drug use (%)	28.42	40.22	0.0004	29.93	38.29	0.012	36.54	23.81	0.001
Frequent sleeping pill use (%)	7.38	31.13	<0.0001	15.40	20.94	0.039	20.79	7.94	<0.0001
Depressive symptoms (%)	12.80	47.93	<0.0001	24.51	33.06	0.007	32.91	12.70	<0.0001
CESD-10 score	5.16 ± 3.93	10.33 ±5.90	<0.0001	6.61 ± 4.98	8.49 ± 6.00	<0.0001	8.11 ± 5.80	5.19 ± 3.71	<0.0001
Body mass index, kg/m^2	30.95 ± 7.28	31.90 ± 8.05	0.074	28.96 ± 6.81	34.42 ± 7.55	<0.0001	31.96 ± 7.77	29.37 ± 6.85	<0.0001
Obesity (%)	50.33	54.55	0.229	34.49	74.66	<0.0001	56.54	37.57	<0.0001
Waist circumference, cm	94.96 ± 18.45	97.37 ± 19.64	0.071	90.24 ± 17.87	103.37 ± 17.86	<0.0001	97.47 ± 18.85	91.95 ± 18.79	<0.0001
Dietary patterns									
AHEI-2010	46.17 ± 9.98	43.93 ±9.93	0.002	46.32 ± 10.27	43.74 ± 9.50	0.0002	44.75 ± 9.98	46.65 ± 10.01	0.022
HEI-2015	59.89 ± 9.16	58.34 ± 9.12	0.016	59.83 ± 9.39	58.41 ± 8.82	0.027	58.99 ± 9.14	59.94 ± 9.23	0.212
aMed Score	4.00 ±1.78	3.82 ± 1.77	0.140	3.89 ± 1.82	3.96 ± 1.73	0.564	3.95 ±1.80	3.83 ± 1.71	0.426

[a.] % or mean ±SD among column total.

[b.] p-value from t-test for continuous variables and Pearson chi-squared for categorical variables.

AHEI: Alternate Healthy Eating Index. HEI: Healthy Eating Index. aMed: alternate Mediterranean. ACS: American Community Survey, 2013 5-year estimates.

MET: Metabolic equivalent of task. CESD-10: Center for Epidemiologic Studies Depression Scale Revised 10 item.

High risk for insomnia defined as > 9 on the Women's Health Initiative Insomnia Rating Scale.

High risk for sleep apnea defined as positive on two of three categories on the Berlin questionnaire.

Healthy sleep pattern determined if healthy pattern on three of five sleep domains: chronotype, duration, insomnia symptoms, snoring, and daytime sleepiness.

Employed includes full or part time employment.

Depressive symptoms defined as CESD-10 ≥ 10.

Frequent sleeping pill use defined as one-two times per week or more.

Obesity defined as body mass index ≥ 30 kg/m^2.

Table S7. Comparison of those included in the analysis with those not meeting inclusion criteria or excluded due to missing on covariates.

	Sample used for analysis [a] *n*=824	Did not meet inclusion or missing on covariates [a] *n*=474	p-value [b]
Demographic characteristics			
Age in years	48.29 ± 5.18	47.88 ± 5.55	0.288
Male (%)	36.17	49.79	<0.0001
Black persons (%)	29.93	42.49	<0.0001
Years of education (%)		(*n*=471)	
Less than high school	10.56	19.32	
High school	34.71	41.61	<0.0001
Some college and higher	54.73	39.07	
Employed (%)	63.35	63.77 (*n*=472)	0.879
Bed partner (%)	62.26	55.56 (*n*=468)	0.018
Household size	2.89 ± 1.45	2.86 ± 1.57 (*n*=473)	0.677
Number of children in the house	0.85 ± 1.08	0.88 ± 1.16	0.596
Neighborhood characteristics (census tract-level)			
ACS: % persons in poverty	26.29 ± 10.96	28.22 ± 10.50 (*n*=455)	0.002
ACS: median income	36364.08 ± 17408.19	33692.75 ± 14701.06 (*n*=455)	0.004
ACS: % households with no vehicle	10.36 ± 6.69	10.93 ± 6.60 (*n*=455)	0.141
ACS: Index of Concentration at the Extremes	-0.05 ± 0.20	-0.09 ± 0.19 (*n*=455)	0.001
ACS: total households	1749.99 ± 775.36	1651.28 ± 576.35 (*n*=455)	0.010
Modified retail food environment index	11.60 ± 9.16	12.37 ± 8.98 (*n*=455)	0.145
Health and lifestyle factors			
Smoking status (%)		(*n*=472)	
Never	53.88	44.28	
Former	22.45	20.13	<0.0001
Current	23.67	35.59	
Current alcohol use (%)	56.07	55.25 (*n*=467)	0.775
Total energy intake, kcal/d	2319.30 ± 1019.58	2424.15 ± 1083.51 (*n*=250)	0.161
Caffeine intake, mg/d	257.84 ± 329.26	251.29 ± 278.27 (*n*=250)	0.755
Physical activity, MET minutes per week	4941.33 ± 5135.69	6117.01 ± 6272.45 (*n*=373)	0.002
Illicit drug use (%)	33.62	30.17	0.201
Frequent sleeping pill use (%)	17.84	16.24	0.464
Depressive symptoms (%)	28.28	34.18	0.026

CESD-10 score	7.44 ± 5.53	8.46 ± 5.95	0.002
Body mass index, kg/m^2	31.37 ± 7.64	31.57 ± 8.09	0.647
Obesity (%)	52.18	50.00	0.448
Waist circumference, cm	96.02 ± 19.01	97.44 ± 19.58	0.200
Sleep outcomes			
High risk for insomnia (%)	44.05	44.94	0.758
High risk for sleep apnea (%)	44.05	41.56	0.383
Healthy sleep pattern (%)	22.94	20.82 (*n*=461)	0.382
Dietary patterns			
AHEI-2010	45.18 ± 10.01	44.53 ± 9.81 (*n*=250)	0.363
HEI-2015	59.21 ± 9.17	59.30 ± 9.31 (*n*=250)	0.894
aMed	3.92 ± 1.78	3.68 ± 1.78 (*n*=250)	0.057

[a.] % or mean ±SD among column total.

[b.] p-value from t-test for continuous variables or Pearson chi-squared test for categorical variables.

ACS: American Community Survey, 2013 5-year estimates. MET: metabolic equivalent of task. CESD-10: Center for Epidemiologic Studies Depression Scale Revised 10 item. AHEI: Alternate Healthy Eating Index. HEI: Healthy Eating Index. aMed: alternate Mediterranean.

Employed includes full or part time employment.

Illicit drug use includes any reported use of crack/cocaine, heroin, methadone/codeine, speed/ecstasy, or LSD/acid/mushrooms/PCP/special K/etc.

Frequent sleeping pill use defined as one-two times per week or more.

Depressive symptoms defined as CESD-10 ≥ 10.

Obesity defined as body mass index ≥ 30 kg/m^2.

High risk for insomnia defined as > 9 on the Women's Health Initiative Insomnia Rating Scale.

High risk for sleep apnea defined as positive on two of three categories on the Berlin questionnaire.

Healthy sleep pattern determined if healthy pattern on three of five sleep domains: chronotype, duration, insomnia symptoms, snoring, and daytime sleepiness.

Table S8. Adjusted prevalence rate ratios for components of Berlin Questionnaire for sleep apnea risk by quintile and per standard deviation increase in Alternate Healthy Eating Index 2010 dietary pattern.

	**Q1**	**Q2** [a]	**Q3** [a]	**Q4** [a]	**Q5** [a]	**p for trend**	**per SD increase** [a]
**AHEI-2010**	*n*=132	*n*=132	*n*=128	*n*=140	*n*=145		SD=10.01
Berlin cat 1-Snoring	1.00	0.89 (0.73, 1.10)	1.08 (0.89, 1.29)	1.00 (0.85, 1.19)	0.80 (0.62, 1.03)	NS	0.97 (0.90, 1.04)
Berlin cat 2-Sleepiness	1.00	0.81 (0.63, 1.04)	0.92 (0.73, 1.16)	0.90 (0.58, 1.40)	0.53 (0.35, 0.81) **	NS	0.83 (0.72, 0.96) *

[a.] Prevalence rate ratios (95% Confidence Interval)

* $p < 0.05$, ** $p < 0.01$, *** $p < 0.001$; NS: $p > 0.05$.

AHEI: Alternate Healthy Eating Index

Models adjusted for: total energy intake, age, sex, race, education (no college, any college or higher), employed (full or part time), bed partner, number of children in house, Index of Concentration at the Extremes (ICE) of census tract, total number of households in census tract, modified retail food environment index for census tract, smoking status (never, current, former), drinking status (current), caffeine intake (mg/d), current illicit drug use (yes/no), frequent sleeping pills use (1-2 times per week or more), depressive symptoms (CESD-10>10), body mass index (kg/m^2), and physical activity (total MET-minutes per week).

Table S9. Adjusted prevalence rate ratios for healthy classification of each component of the Healthy Sleep Pattern by quintile and per standard deviation increase in Alternate Healthy Eating Index 2010 dietary pattern.

AHEI-2010	Q1 *n*=132	Q2 [a] *n*=132	Q3 [a] *n*=128	Q4 [a] *n*=140	Q5 [a] *n*=145	p for trend	per SD increase [a] SD=10.01
HSP 1: Chronotype	1.00	1.05 (0.88, 1.25)	0.97 (0.74, 1.28)	1.05 (0.85, 1.31)	1.06 (0.83, 1.36)	NS	1.03 (0.94, 1.11)
HSP 2: Duration	1.00	1.04 (0.88, 1.23)	1.07 (0.79, 1.46)	1.07 (0.87, 1.31)	1.10 (0.87, 1.41)	NS	1.04 (0.97, 1.12)
HSP 3: Insomnia symptoms	1.00	1.03 (0.73, 1.44)	1.08 (0.67, 1.72)	1.06 (0.68, 1.65)	1.22 (0.71, 2.10)	NS	1.10 (0.93, 1.31)
HSP 4: Snoring	1.00	0.99 (0.74, 1.33)	0.88 (0.72, 1.08)	1.11 (0.89, 1.37)	1.13 (0.92, 1.37)	NS	1.04 (0.97, 1.12)
HSP 5: Sleepiness	1.00	1.02 (0.91, 1.13)	1.03 (0.92, 1.16)	1.04 (0.92, 1.17)	1.01 (0.92, 1.11)	NS	1.00 (0.97, 1.03)

[a.] Prevalence rate ratios (95% Confidence Interval)

[*] $p < 0.05$, [**] $p < 0.01$, [***] $p < 0.001$; NS: $p > 0.05$.

AHEI: Alternate Healthy Eating Index

HSP 1-Chronotype: Healthy defined as "morning type" or "more morning than evening type" based on the reduced Morningness-Eveningness Questionnaire.

HSP 2-Duration: healthy if average self-report sleep duration ≥7 hours and <9 hours.

HSP 3-Insomnia symptoms: healthy if no frequent insomnia symptoms, from the Women's Health Initiative Insomnia Rating Scale.

HSP 4-Snoring: healthy if no snoring in the past 4 weeks, from the Berlin Questionnaire.

HSP 5-Sleepiness: healthy if score <11 on the Epworth Sleepiness Scale.

Models adjusted for: total energy intake, age, sex, race, education (no college, any college or higher), employed (full or part time), bed partner, number of children in house, Index of Concentration at the Extremes (ICE) of census tract, total number of households in census tract, modified retail food environment index for census tract, smoking status (never, current, former), drinking status (current), caffeine intake (mg/d), current illicit drug use (yes/no), frequent sleeping pills use (1-2 times per week or more), depressive symptoms (CESD-10>10), body mass index (kg/m^2), and physical activity (total MET-minutes per week).

Table S10. Adjusted prevalence rate ratios for sleep outcomes by quintile and per standard deviation increase in Alternate Healthy Eating Index 2010 dietary pattern, among those without depressive symptoms (CESD-10 < 10). (n=591).

	**Q1**	**Q2 [a]**	**Q3 [a]**	**Q4 [a]**	**Q5 [a]**	**p for trend**	**per SD increase [a]**
**AHEI-2010**	*n*=104	*n*=113	*n*=112	*n*=126	*n*=136		SD=10.01
Insomnia risk	1.00	0.97 (0.66, 1.44)	0.79 (0.55, 1.14)	0.80 (0.58, 1.10)	0.72 (0.50, 1.02)	0.016	0.89 (0.80, 1.00) *
Sleep apnea risk	1.00	0.76 (0.58, 1.00) *	0.97 (0.80, 1.18)	0.82 (0.69, 0.99) *	0.59 (0.44, 0.81) ***	0.001	0.90 (0.82, 0.99) *
Healthy sleep pattern	1.00	1.13 (0.71, 1.78)	0.88 (0.52, 1.47)	1.05 (0.76, 1.47)	1.19 (0.77, 1.85)	NS	1.06 (0.93, 1.22)

[a.] Prevalence Rate Ratio (95% Confidence Interval)

* $p < 0.05$, ** $p < 0.01$, *** $p < 0.001$; NS: $p > 0.05$.

AHEI: Alternate Healthy Eating Index

High risk for insomnia defined as > 9 on the Women's Health Initiative Insomnia Rating Scale.

High risk for sleep apnea defined as positive on two of three categories on the Berlin questionnaire.

Healthy sleep pattern determined if healthy pattern on three of five sleep domains: chronotype, duration, insomnia symptoms, snoring, and daytime sleepiness.

Models adjusted for: total energy intake, age, sex, race, education (no college, any college or higher), employed (full or part time), bed partner, number of children in house, Index of Concentration at the Extremes (ICE) of census tract, total number of households in census tract, modified retail food environment index (mRFEI) for census tract, smoking status (never, current, former), drinking status (current), caffeine intake (mg/d), current illicit drug use (yes/no), frequent sleeping pills use (1-2 times per week or more), depressive symptoms (CESD-10>10), body mass index (kg/m^2), and physical activity (total MET-minutes per week).

Table S11. Adjusted prevalence rate ratios for sleep outcomes by quintile and per standard deviation increase in Alternate Healthy Eating Index 2010 dietary pattern, among those who do not use sleeping pills frequently (< once per week). (n=677).

**AHEI-2010**	**Q1** *n*=132	**Q2** [a] *n*=132	**Q3** [a] *n*=128	**Q4** [a] *n*=140	**Q5** [a] *n*=145	**p for trend**	**per SD increase** [a] SD=10.01
Insomnia risk	1.00	1.05 (0.81, 1.36)	0.88 (0.62, 1.23)	0.95 (0.74, 1.21)	0.87 (0.65, 1.16)	NS	0.95 (0.88, 1.03)
Sleep apnea risk	1.00	0.74 (0.59, 0.92) **	0.89 (0.77, 1.03)	0.79 (0.65, 0.97) *	0.60 (0.44, 0.81) **	0.0003	0.89 (0.81, 0.97) **
Healthy sleep pattern	1.00	1.04 (0.68, 1.59)	1.02 (0.62, 1.68)	1.06 (0.78, 1.45)	1.24 (0.81, 1.91)	NS	1.08 (0.94, 1.23)

[a.] Prevalence rate ratios (95% Confidence Interval)

* $p < 0.05$, ** $p < 0.01$, *** $p < 0.001$; NS: $p > 0.05$.

AHEI: Alternate Healthy Eating Index

High risk for insomnia defined as > 9 on the Women's Health Initiative Insomnia Rating Scale.

High risk for sleep apnea defined as positive on two of three categories on the Berlin questionnaire.

Healthy sleep pattern determined if healthy pattern on three of five sleep domains: chronotype, duration, insomnia symptoms, snoring, and daytime sleepiness.

Models adjusted for: total energy intake, age, sex, race, education (no college, any college or higher), employed (full or part time), bed partner, number of children in house, Index of Concentration at the Extremes (ICE) of census tract, total number of households in census tract, modified retail food environment index for census tract, smoking status (never, current, former), drinking status (current), caffeine intake (mg/d), current illicit drug use (yes/no), frequent sleeping pills use (1-2 times per week or more), depressive symptoms (CESD-10>10), body mass index (kg/m^2), and physical activity (total MET-minutes per week).

References (also referenced in main text)

1. Chiuve, S.E.; Fung, T.T.; Rimm, E.B.; Hu, F.B.; McCullough, M.L.; Wang, M.; Stampfer, M.J.; Willett, W.C. Alternative Dietary Indices Both Strongly Predict Risk of Chronic Disease. *The Journal of Nutrition* **2012**, *142*, 1009–1018, doi:10.3945/jn.111.157222.
2. Reedy, J.; Lerman, J.L.; Krebs-Smith, S.M.; Kirkpatrick, S.I.; Pannucci, T.R.E.; Wilson, M.M.; Subar, A.F.; Kahle, L.L.; Tooze, J.A. Evaluation of the Healthy Eating Index-2015. *Journal of the Academy of Nutrition and Dietetics* **2018**, *118*, 1622–1633, doi:10.1016/j.jand.2018.05.019.
3. Fung Teresa T.; Rexrode Kathryn M.; Mantzoros Christos S.; Manson JoAnn E.; Willett Walter C.; Hu Frank B. Mediterranean Diet and Incidence of and Mortality From Coronary Heart Disease and Stroke in Women. *Circulation* **2009**, *119*, 1093–1100, doi:10.1161/CIRCULATIONAHA.108.816736.
4. Levine, D.W.; Lewis, M.A.; Bowen, D.J.; Kripke, D.F.; Kaplan, R.M.; Naughton, M.J.; Shumaker, S.A. Reliability and Validity of Women's Health Initiative Insomnia Rating Scale. *Psychological Assessment* **2003**, *15*, 137–148, doi:10.1037/1040-3590.15.2.137.
5. Netzer, N.C.; Stoohs, R.A.; Netzer, C.M.; Clark, K.; Strohl, K.P. Using the Berlin Questionnaire To Identify Patients at Risk for the Sleep Apnea Syndrome. *Annals of Internal Medicine* **1999**, *131*, 485, doi:10.7326/0003-4819-131-7-199910050-00002.
